# Vaccine hesitancy and related factors among South African adults in 2021: unpacking uncertainty versus unwillingness

**DOI:** 10.3389/fpubh.2023.1233031

**Published:** 2023-11-03

**Authors:** Ronel Sewpaul, Sibusiso Sifunda, Razia Gaida, Tholang Mokhele, Inbarani Naidoo, Sasiragha Priscilla Reddy

**Affiliations:** ^1^Public Health, Societies and Belonging, Human Sciences Research Council, Pretoria, South Africa; ^2^Aquity Innovations, Port Elizabeth, South Africa; ^3^eResearch Knowledge Centre, Human Sciences Research Council, Pretoria, South Africa; ^4^College of Humanities, University of KwaZulu-Natal, Durban, South Africa

**Keywords:** COVID-19 vaccinations, vaccine hesitancy, trust, side-effects, influenza vaccination, South Africa

## Abstract

**Background:**

Amidst widespread public health recommendations and availability of COVID-19 vaccinations, half of South African adults are vaccinated against COVID-19. This study investigated the socio-behavioral determinants of vaccine hesitancy in South Africa, where vaccine hesitancy was separated into unwilling ness and uncertainty to take a COVID-19 vaccine.

**Methods:**

Data was collected from a large-scale public survey during June–October 2021 that included online and telephonic surveys. Vaccination hesitancy was based on the question “When available, would you take the COVID 19 vaccine?,” with responses categorized into those who were willing, unwilling, and uncertain about taking a COVID-19 vaccine. Multinomial regression examined the association between socio-behavioral variables and vaccine hesitancy.

**Results:**

Overall, 73.8% reported they would definitely or probably take the vaccine, 16.4% were uncertain and 9.9% reported they probably or definitely would not (*n* = 16,988). Younger age, White and Colored population groups, no influenza vaccination history, previous vaccination refusal, knowing someone who experienced a serious vaccination side-effect, misperceptions about vaccine benefits, cultural or religious discouragement from taking a COVID-19 vaccination, lack of governmental confidence, concerns about side-effects, perceived lack of safety information, and lack of trust in the pharmaceutical industry and in the information from health care providers were all associated with higher odds of being uncertain and unwilling to take a COVID-19 vaccination. Strengths of association for unwillingness and uncertainty varied by the explanatory variables. Concern about effectiveness due to fast development was associated with uncertainty to take the vaccine but not with unwillingness. Concerns about side-effects had stronger associations with uncertainty than with unwillingness, while previous vaccine refusal, misperceptions of the protective benefits of vaccines, White population group, religious/cultural discouragement, and lack of trust in the pharmaceutical industry and health care providers’ information had stronger associations with unwillingness than uncertainty.

**Conclusion:**

The determinants of COVID-19 vaccine hesitancy should be addressed in interventions to improve vaccine uptake. Public health interventions and health communication can be prioritized and tailored to the different forms of vaccination hesitancy.

## Introduction

Vaccine hesitancy, defined by the World Health Organization as the “delay in acceptance or refusal of vaccination despite availability of vaccination services,” was among the top 10 global health threats in 2019 (1). There is a continuum of vaccine hesitancy between complete acceptance and absolute refusal of all vaccines (2).

Despite COVID-19 vaccinations being one of the most effective strategies to control the pandemic, vaccination acceptance and uptake rates vary significantly globally (3). By January 2023, 64% of people worldwide were fully vaccinated against COVID-19 and 70% had received at least one vaccine dose (4, 5), with the African continent having far lower vaccination coverage (4). An in-depth understanding of the socio-behavioral determinants of COVID-19 vaccination hesitancy and the characteristics of people who are more likely to be uncertain about or refuse a COVID-19 vaccine is required for comprehensive and effective vaccination strategies. Interventions to improve vaccine uptake can be tailored to the various positions held along the vaccine acceptance continuum.

South Africa’s national vaccination program began in February 2021 with a staggered approach starting with healthcare workers and then age groups from oldest to youngest eligible. Amidst public health recommendations and wide availability of COVID-19 vaccinations in the country, only half of South African adults and 35% of children aged 12–17 had been vaccinated by March 2023 (6). Adults aged 30 or older and females had higher vaccination rates than young adults and males. Vaccination rates have stagnated since mid-2022.

Vaccine hesitancy, in general, is influenced by an array of cognitive, socio-demographic, psychologic, political and cultural factors (7). The literature on factors associated with COVID-19 vaccine hesitancy identifies issues around vaccine safety and efficacy, risk perceptions of COVID-19 infection and its severity, and concerns about the rapid development of the COVID-19 vaccines as prominent factors (7–13). Given that the COVID-19 vaccines were developed faster than other vaccines in the past, this has evidently contributed to hesitancy (7). Issues of trust play a key role in regulating vaccine acceptance, including trust in governments, health professionals, scientists and the pharmaceutical industry (14, 15). Sources of information about the pandemic and vaccinations (16) are another contributing factor. Misinformation and disinformation can diminish vaccine acceptance (17, 18). The flood of information about the pandemic and COVID-19 vaccines, often termed the COVID “infodemic,” included both factual and misleading or false information, that complicates access to reliable information about vaccination (19). Poor influenza-vaccination history also resulted in hesitancy to take a COVID-19 vaccination in several studies (11, 15, 18). In terms of socio-demographic factors, vaccine hesitancy was found to be higher in individuals with lower education levels and awareness, minority race groups, younger age groups, some women, and lower income groups (7, 15, 18).

This study investigated the socio-behavioral factors associated with COVID-19 vaccine hesitancy among South African adults aged 18 years and older during the third quarter of 2021, using data from a national survey. While the majority of studies on vaccine hesitancy in South Africa and internationally have focused on vaccine hesitancy as a homogenous group, this study aims to bridge that gap by separating hesitancy into individuals who were unwilling or did not want to be vaccinated and those who were uncertain about their intentions to be vaccinated. It was hypothesized that the identified factors and their strengths of association would differ for those who were unwilling versus those who were uncertain to receive the vaccination.

## Materials and methods

### Study design and setting

Data were collected using an online survey questionnaire and telephone facilitated interviews between 25 June and 11 October 2021. South Africans aged ≥18 years in all nine provinces were eligible to participate. During this data collection period, the country was experiencing its third wave of COVID-19 infections, with higher hospitalization rates than the previous two waves (20). A strict lockdown was implemented during July 2021. The country’s vaccination program was also underway, where vaccinations became available to persons aged 60 and older from mid-May 2021, to those aged 50–59 by July 2021, to those aged 35–49 from 1 August 2021; and by the end of August 2021, vaccinations were available to all adults aged 18 and older. Children aged 12 and older followed from October 2021. Furthermore, some employment categories such as educators and police service employees were prioritized during June and July 2021 (21).

Information about the survey and the invitation to participate were distributed on a data-free mobile messaging platform as well as on social media platforms, national and local radio, national television, local websites and communication networks in government, education, faith-based and community organizations, non-profit organizations and private sector organizations. The data-free mobile messaging platform has a substantial user-base of over 4 million South Africans, and it is accessible from most application stores. The data-free platform facilitated survey completion without incurring user data costs. This methodology was used previously by the authors (22, 23). The questionnaire and telephonic interviews were available to complete in six of South Africa’s languages; namely, English, Afrikaans, isiZulu, TshiVenda, xiTsonga and isiXhosa.

Telephonic interviews supplemented the online survey approach in order to include participants that would not typically participate in online surveys. Interns employed by the Human Sciences Research Council were selected to work as telephone interviewers. The interns were aged between 20 and 35 years, had attained a graduate degree and were enrolled in an experiential training program in social sciences research at the Human Sciences Research Council (HSRC). The interviewers were trained in procedures for obtaining informed consent and administering telephone interviews. The interviewers were collectively fluent in the six languages in which the survey could be completed. An anonymized list of telephone numbers of over 1 million people residing in predominantly densely populated areas such as informal settlements and townships (urban residential settlements) was used to recruit participants in the telephone survey, where 16,000 people from the list were selected and telephonically contacted and 7,962 participated.

### Questionnaire development

The questionnaire was developed by a research team at the HSRC that constituted epidemiologists, and public health and behavioral scientists. The questionnaire development was informed by previous public online survey research conducted by the HSRC research team during the emergence of the COVID-19 pandemic (22–25) as well as previous a multi-country survey (26), and the questionnaire development was conducted in consultation with the South African National Department of Science and Technology. The questionnaire was refined using inputs from stakeholders in scientific and civil society networks. The thematic areas included in the questionnaire covered demographic characteristics, attitudes and experiences of vaccinations; culture, context and communications regarding the COVID-19 vaccinations, opinions on South Africa’s vaccination plan, grief and coping during the pandemic, social distancing behavior, the socio-economic impact of COVID-19, and family relationship dynamics during the COVID-19 pandemic. The questionnaire items were predominantly close ended.

### Measures

The primary outcome variable, COVID-19 vaccination hesitancy, was derived from the question “When available, would you take the COVID 19 vaccine?” with five possible response options 1 = “Yes definitely, I would take the vaccine,” 2 = “Yes probably, I would take the vaccine,” 3 = “I am uncertain at this stage,” 4 = “No, probably I would NOT take the vaccine” and 5 = “No definitely, I would NOT take the vaccine.” The responses were recoded into three categories where 1 = “Willing: Would take the vaccine” (coded from options 1 and 2 above); 2 = “Uncertain” and 3 = “Unwilling: Would not take the vaccine” (coded from options 4 and 5 above).

The selection of explanatory variables was informed by the literature on COVID-19 vaccine hesitancy [7–16]. The explanatory variables used can be categorized as *socio-demographic* (gender, age group, population group, province, community type, educational attainment, employment), *experiences and perceptions related to other vaccines or vaccines in general* (having ever taken the influenza vaccine, having ever refused to take any vaccine, knowing anyone who experienced a serious side-effect to any vaccine, thinking that vaccines are a good way to protect communities from disease), *information on the COVID-19 vaccinations* (main source of information on the COVID-19 vaccinations, having heard conflicting or confusing information about the COVID-19 vaccinations), *religious/cultural influence* (feeling that one’s religion or culture would discourage one from receiving a COVID-19 vaccination), *trust/confidence in governments and health and scientific institutions* (level of confidence in how the national government was handling the pandemic, trust in the pharmaceutical industry with developing the COVID-19 vaccine, trust in the information from one’s health care provider about the COVID-19 vaccinations), *perceptions of risk, safety and efficacy* (concerned about side-effects related to the COVID-19 vaccines, thinking that there is adequate safety information related to the COVID-19 vaccination program, perception that the effectiveness of the COVID-19 vaccine will be in question due to its fast development and having lost anyone close to you during the lockdown period).

The questions from which these variables are based are presented in Supplementary File S1. Population group was reported in alignment with Statistics South Africa’s standard classification categories (27).

### Statistical analysis

Data were analyzed on participants who responded to the question on vaccine hesitancy. Analyses were performed using Stata 15.0 (Stata Corporation, College Station, Texas, United States). The data were benchmarked using the South African adult mid-year population estimates for 2021 by age, sex, population group and province (27) in order to increase generalizability of the estimates at national level. The percentages of participants who did want to receive a COVID-19 vaccine, who were uncertain at the time, and who did not want to receive the vaccine were tabulated by the explanatory variables of interest, with Pearson Chi-square tests used to identify significant differences in estimates.

Multivariate multinomial logistic regression was used to determine the explanatory variables associated with being uncertain and unwilling to receive the COVID-19 vaccine, where “would take the vaccine” was used as the reference category. The explanatory variables that exhibited a significant univariate association with the outcome variable, as determined by Chi-square tests, were included in the multivariate multinomial model. Pairwise correlations between the explanatory variables were used to check for multicollinearity. However, all pairwise correlation coefficients were less than 0.35. Odds ratios and their 95% confidence intervals measured the strength and direction of the associations in the multinomial regression model. All statistical tests were considered significant at *p* < 0.05.

## Results

### Description of the weighted sample

The sample comprised 52% females and the mean age was 40.1 years (Standard. error = 0.355) (Table 1). Over three-quarters (77.2%) identified as Black African. Over 64% had completed secondary school and 56.8% were employed. Regarding previous experiences with vaccines in general, almost a third (31.8%) had ever taken an influenza vaccination, 13.0% had ever refused to receive any vaccination, 22.1% had objected to allow someone else to receive a vaccination, 27.7% knew someone who had experienced a serious side-effect to any vaccine and 64.4% thought that vaccines were a good way to protect communities from disease. The most prevalent responses to the main source of information on the COVID-19 vaccine and vaccinations were television (46.6%), followed by radio (17.2%) and social media (11.0%). Almost three in four people (72.4%) reported hearing conflicting or confusing information about the COVID-19 vaccine and vaccinations and 16.7% felt that their religion or culture would discourage them from taking a COVID-19 vaccine. Moderate or high confidence in the government’s handling of the pandemic was reported by 68.1% of participants, while 69.9% trusted the information from their health care providers about the COVID-19 vaccinations and 53.2% trusted the pharmaceutical industry with developing the vaccine. Over 60% were concerned about side-effects from the COVID-19 vaccine, 48.0% thought there was adequate safety information about the COVID-19 vaccination program and 49.8% thought the effectiveness of the vaccinations would be in question due to their fast development.

**Table 1:** Description of the weighted sample.

	%	95% CI	N
Total	100.0		16,988
Socio-demographic			
Gender			
Female	52.0	[50.7–53.4]	9,544
Male	48.0	[46.6–49.3]	7,119
Other	0.0	[0.0–0.0]	319
Age group (years)			
18–29	28.4	[27.6–29.3]	8,242
30–39	26.5	[25.6–27.4]	4,866
40–49	18.6	[17.7–19.5]	1,848
50–59	12.7	[11.8–13.8]	658
60–69	9.1	[7.9–10.3]	220
70+	4.7	[3.4–6.3]	42
Population group			
Black African	77.2	[75.9–78.6]	13,274
White	10.2	[9.0–11.6]	500
Colored	9.4	[8.9–9.9]	2,238
Indian/Asian	3.1	[2.6–3.6]	327
Prefer not to answer	0.1	[0.1–0.2]	648
Province			
Western Cape	12.7	[12.0–13.4]	2,277
Eastern Cape	10.2	[9.5–11.0]	1,437
Northern Cape	2.1	[1.8–2.5]	437
Free State	4.9	[4.5–5.3]	1,242
KwaZulu-Natal	17.2	[16.1–18.2]	2,544
North-West	7.0	[6.5–7.6]	1,049
Gauteng	30.0	[29.0–31.0]	5,334
Mpumalanga	7.5	[7.0–8.1]	1,025
Limpopo	8.4	[7.7–9.2]	1,643
Community type			
City	11.0	[10.1–11.8]	1,925
Suburb	17.1	[15.9–18.4]	2,316
Township	44.0	[42.7–45.3]	7,629
Informal settlement	7.3	[6.7–7.9]	1,374
Rural (traditional tribal area)	18.3	[17.3–19.3]	3,185
Farm	2.4	[2.0–2.9]	418
Highest educational attainment			
Less than secondary school	9.3	[8.2–10.4]	1,023
Secondary school	26.4	[25.3–27.6]	4,437
Matric (grade 12 school level)	40.6	[39.3–41.9]	7,360
Tertiary	23.7	[22.6–24.9]	3,753
Employment status			
Employed	32.1	[30.9–33.3]	4,677
Unemployed	56.8	[55.4–58.2]	10,584
Not in labor force	11.1	[9.8–12.5]	1,316
Experiences and perceptions related to other vaccines or vaccines in general			
Ever taken the FLU vaccine			
Yes	31.8	[30.5–33.2]	4,801
No	68.2	[66.8–69.5]	12,041
Ever personally refused to take any vaccine			
Yes	13.0	[12.2–13.9]	2,320
No	87.0	[86.1–87.8]	14,550
Ever objected to allow someone else to take a vaccine			
Yes	22.1	[21.1–23.1]	4,408
No	77.9	[76.9–78.9]	12,443
Know anyone who has personally experienced a serious side-effect to any vaccine			
Yes	27.7	[26.6–28.9]	5,159
No	72.3	[71.1–73.4]	11,706
Perceive vaccines as a good way to protect communities from disease			
Yes	64.4	[63.2–65.6]	9,988
No	5.3	[4.8–5.9]	990
Not sure	30.2	[29.1–31.4]	5,895
Information on the COVID-19 vaccinations			
Main source of information on the COVID-19 vaccine and vaccinations			
Television	46.6	[45.2–48.0]	7,067
Radio	17.2	[16.1–18.4]	2,335
News sources (print or online)	8.2	[7.5–8.9]	1,247
Government sources	7.6	[6.9–8.4]	1,142
Medical sources	5.0	[4.2–6.0]	550
Social media	11.0	[10.2–11.7]	2,008
Other	4.4	[3.9–4.9]	685
Heard conflicting/confusing information related to the COVID19 vaccine and vaccinations			
Yes	72.4	[71.1–73.6]	10,857
No	27.6	[26.4–28.9]	4,062
Religious/cultural influence			
Feel that your religion or culture would discourage you from getting a COVID-19 vaccine			
Yes	16.7	[15.7–17.7]	2,580
No	83.3	[82.3–84.3]	10,436
Trust/confidence in governments and health and scientific institutions			
Confidence in how the national government is handling the pandemic			
Very confident	42.3	[40.7–43.8]	4,940
Moderately confident	25.8	[24.5–27.1]	3,341
Not confident	17.6	[16.5–18.8]	2,352
I have no opinion	14.3	[13.4–15.3]	2,119
Trust in the information from your health care provider about the COVID-19 vaccinations			
Yes	69.9	[68.5–71.2]	8,755
No	8.8	[8.0–9.6]	1,206
Not sure	21.4	[20.2–22.6]	2,943
Trust in the pharmaceutical industry with developing the COVID-19 vaccine			
Yes	53.2	[51.7–54.7]	6,385
No	12.8	[11.9–13.8]	1,807
Not sure	34.0	[32.6–35.4]	4,731
Perceptions of risk, safety and efficacy			
Concerned about any side-effects related to the COVID-19 vaccines			
Yes	61.2	[59.8–62.6]	11,120
No	23.8	[22.5–25.1]	3,203
Not sure	15.0	[14.1–15.9]	2,610
Think there is adequate safety information related to the COVID-19 vaccination program			
Yes	48.0	[46.3–49.7]	5,050
No	22.2	[20.7–23.8]	2,379
Not sure	29.8	[28.3–31.3]	3,524
Believe the effectiveness of the COVID-19 vaccine will be in question due to its fast development			
Yes	49.8	[48.1–51.5]	5,966
No	20.9	[19.4–22.6]	1,786
Not sure	29.3	[27.8–30.8]	3,185
Lost anyone close to you during the lockdown period			
Yes	55.1	[53.4–56.8]	5,901
No	44.9	[43.2–46.6]	4,772

### Vaccine hesitancy by explanatory variables

Overall, 74.5% of the eligible participants reported that they would take a COVID-19 vaccine, 15.7% were uncertain at the time and 9.9% would not take the vaccine.

The percentages of participants who were willing, uncertain and unwilling to take a vaccine varied significantly with all the explanatory variables (Table 2). We highlight the characteristics of people who were uncertain or unwilling to vaccinate. Vaccine hesitancy, that is, the prevalence of being uncertain or unwilling to take a vaccine, were both higher among 18–29-year-olds, those who had never received an influenza vaccination, those who had previously refused to take a vaccine, who knew someone who experienced a vaccine-related side-effect, who heard conflicting information about the COVID-19 vaccinations, who were not confident in the government’s handling of the pandemic, who did not trust or were unsure of their trust in the pharmaceutical industry with vaccine development and in the information from their health care providers, who felt religious or cultural discouragement about the vaccinations, and among those who had concerns about side-effects, safety and effectiveness.

**Table 2:** Uncertainty and willingness to access the COVID-19 vaccine by socio-demographic variables.

	When available, would you take the COVID-19 vaccine?						
	Would take the vaccine		Uncertain		Would not take the vaccine		*p*-value
	Row %	95% CI	Row %	95% CI	Row %	95% CI	
Total	74.5	[73.4–75.5]	15.7	[14.9–16.5]	9.9	[9.2–10.6]	
Gender							<0.001
Female	71.8	[70.3–73.3]	18	[16.8–19.3]	10.1	[9.2–11.2]	
Male	77.3	[75.8–78.7]	13.1	[12.1–14.2]	9.5	[8.5–10.7]	
Other	61.6	[55.7–67.1]	19.2	[15.0–24.3]	19.2	[15.0–24.3]	
Age group							<0.001
18–29	65.1	[64.0–66.2]	22.3	[21.3–23.2]	12.6	[11.8–13.4]	
30–39	73.3	[72.0–74.7]	16.8	[15.7–17.9]	9.9	[9.0–10.9]	
40–49	76.6	[74.3–78.7]	13.9	[12.2–15.8]	9.5	[8.1–11.2]	
50–59	80.2	[76.4–83.5]	11.7	[9.3–14.8]	8.1	[5.8–11.2]	
60–69	88.1	[82.1–92.2]	5.4	[2.9–9.9]	6.6	[3.6–11.7]	
≥70	87.2	[73.7–94.3]	7.4	[2.4–20.7]	5.4	[1.7–15.9]	
Population group							<0.001
Black African	76.8	[75.8–77.8]	14.9	[14.1–15.8]	8.3	[7.7–9.0]	
White	65.8	[59.5–71.5]	15	[11.5–19.4]	19.2	[14.9–24.4]	
Colored	63.8	[61.2–66.3]	23.7	[21.6–25.9]	12.5	[10.9–14.3]	
Indian/Asian	76.8	[70.5–82.1]	13.9	[9.9–19.1]	9.3	[6.2–13.7]	
Prefer not to answer	73.1	[38.2–92.3]	0.3	[0.2–0.6]	26.5	[7.5–61.8]	
Province							0.001
Western Cape	70.9	[67.9–73.7]	18.8	[16.7–21.1]	10.3	[8.4–12.6]	
Eastern Cape	76.4	[72.6–79.8]	15.4	[12.4–19.1]	8.2	[6.5–10.2]	
Northern Cape	71.5	[65.2–77.1]	18.1	[13.9–23.2]	10.4	[7.4–14.4]	
Free State	73.1	[68.8–77.0]	13.4	[11.1–16.1]	13.5	[10.1–17.8]	
KwaZulu-Natal	73.4	[70.4–76.3]	16.8	[14.5–19.4]	9.8	[8.1–11.7]	
North-West	72	[67.7–76.0]	16	[13.4–19.0]	12	[8.8–16.1]	
Gauteng	75.7	[74.0–77.4]	14.7	[13.4–16.0]	9.6	[8.5–10.8]	
Mpumalanga	71.1	[67.2–74.7]	18.6	[15.6–22.1]	10.3	[8.2–12.7]	
Limpopo	81.6	[77.8–84.8]	10.5	[8.8–12.5]	8	[5.3–11.8]	
Community type							0.004
City	75.1	[71.7–78.3]	14.6	[12.0–17.6]	10.3	[8.4–12.5]	
Suburb	73.5	[70.2–76.5]	14.9	[13.0–17.0]	11.6	[9.4–14.3]	
Township	73.6	[72.1–75.0]	16.3	[15.2–17.4]	10.2	[9.2–11.2]	
Informal settlement	72.6	[68.5–76.4]	19.4	[15.9–23.3]	8	[6.2–10.3]	
Rural (traditional tribal area)	79	[76.9–81.0]	13.9	[12.3–15.6]	7.1	[5.9–8.6]	
Farm	67.1	[56.6–76.2]	18.5	[11.7–28.2]	14.3	[7.8–24.8]	
Educational attainment							0.001
Less than secondary	79	[73.7–83.5]	9	[6.1–13.0]	12	[8.7–16.5]	
Secondary	75.7	[73.7–77.6]	15	[13.6–16.7]	9.3	[8.1–10.6]	
Matric	72.2	[70.6–73.7]	17.5	[16.3–18.7]	10.3	[9.3–11.5]	
Tertiary	75.3	[73.1–77.4]	15.8	[14.2–17.6]	8.9	[7.6–10.4]	
Employment status							0.002
Employed	76.3	[74.4–78.0]	13.8	[12.5–15.3]	9.9	[8.7–11.3]	
Unemployed	72.5	[71.2–73.8]	17.4	[16.4–18.4]	10.1	[9.2–11.1]	
Not in labor force	79.5	[74.8–83.5]	12.2	[9.3–15.9]	8.3	[5.8–11.7]	
Ever taken the flu vaccine							<0.001
Yes	82.3	[80.5–84.0]	10.5	[9.4–11.8]	7.1	[5.9–8.6]	
No	70.7	[69.4–72.0]	18.1	[17.1–19.2]	11.2	[10.3–12.1]	
Ever refused to take any vaccine							<0.001
Yes	50.8	[47.3–54.3]	20.8	[18.2–23.6]	28.4	[25.5–31.4]	
No	78	[76.9–79.1]	14.9	[14.1–15.8]	7.1	[6.4–7.8]	
Know anyone who has experienced a serious side-effect to any vaccine							<0.001
Yes	62.6	[60.5–64.6]	20.5	[18.9–22.1]	16.9	[15.5–18.5]	
No	78.9	[77.7–80.1]	13.9	[13.0–14.9]	7.2	[6.4–8.1]	
Perceive vaccines as a good way to protect communities from disease							<0.001
Yes	89	[88.0–89.9]	7.8	[7.0–8.7]	3.2	[2.7–3.8]	
No	29.2	[24.7–34.1]	16.7	[13.6–20.3]	54.2	[49.1–59.2]	
Not sure	51.3	[49.1–53.5]	32.5	[30.6–34.4]	16.2	[14.7–17.9]	
Main source of information on the COVID19 vaccine and vaccinations							<0.001
Television	76.3	[74.7–77.8]	15.1	[14.0–16.3]	8.6	[7.6–9.7]	
Radio	78.3	[75.3–81.0]	14.7	[12.4–17.4]	7	[5.5–8.7]	
News sources (print or online)	71.2	[67.4–74.8]	17.9	[15.3–21.0]	10.8	[8.4–13.9]	
Government sources	73.4	[69.0–77.3]	17.5	[14.1–21.6]	9.1	[7.1–11.7]	
Medical sources	73.9	[66.0–80.5]	12.9	[9.3–17.7]	13.2	[8.1–20.7]	
Social media	70.6	[67.7–73.3]	18.3	[16.3–20.6]	11.1	[9.2–13.3]	
Other	60.2	[54.6–65.5]	16.4	[13.1–20.3]	23.4	[18.8–28.8]	
Heard conflicting/confusing information about the COVID-19 vaccinations							<0.001
Yes	72.5	[71.1–73.8]	17.2	[16.2–18.3]	10.3	[9.4–11.3]	
No	79.6	[77.7–81.5]	12.4	[11.0–14.0]	7.9	[6.7–9.3]	
Feel that your religion or culture would discourage you from getting a COVID-19 vaccination							<0.001
Yes	62.9	[59.6–66.0]	19.7	[17.0–22.6]	17.4	[15.1–20.1]	
No	77	[75.7–78.2]	15.3	[14.3–16.3]	7.7	[7.0–8.6]	
Confidence in how the national government is handling the pandemic							<0.001
Not confident	53.2	[49.5–56.8]	23.6	[21.2–26.2]	23.2	[20.4–26.3]	
No opinion	60.6	[57.3–63.8]	24.6	[22.0–27.5]	14.7	[12.6–17.1]	
Moderately confident	76.6	[74.4–78.6]	17.2	[15.5–18.9]	6.3	[5.0–7.8]	
Very confident	86.8	[85.0–88.3]	9.6	[8.2–11.1]	3.7	[3.0–4.6]	
Concerned about any side-effects related to the COVID-19 vaccines?							<0.001
Yes	67.4	[65.9–68.8]	20.6	[19.5–21.8]	12	[11.0–13.1]	
No	91.2	[89.7–92.4]	3.5	[2.8–4.2]	5.4	[4.3–6.6]	
Not sure	77	[74.5–79.2]	14.9	[13.1–16.9]	8.2	[6.7–9.8]	
Trust in the pharmaceutical industry with developing the COVID-19 vaccine							<0.001
Yes	91.2	[90.0–92.2]	6.6	[5.7–7.7]	2.2	[1.8–2.6]	
No	37.7	[33.8–41.9]	22.7	[20.1–25.5]	39.5	[35.8–43.5]	
Not sure	62.4	[60.1–64.6]	28.5	[26.6–30.6]	9.1	[7.9–10.5]	
Trust in the information from your health care provider about the COVID-19 vaccinations							<0.001
Yes	85.3	[84.2–86.4]	10.8	[9.9–11.8]	3.9	[3.3–4.5]	
No	37.3	[33.0–41.9]	20.5	[17.5–23.8]	42.2	[37.7–46.8]	
Not sure	54.7	[51.6–57.8]	31.6	[28.9–34.4]	13.7	[11.8–15.9]	
Think there is adequate safety information related to the COVID-19 vaccination program							<0.001
Yes	87.7	[86.4–88.9]	8.6	[7.6–9.6]	3.7	[3.0–4.5]	
No	57.7	[53.9–61.4]	20.7	[18.0–23.6]	21.6	[18.9–24.6]	
Not sure	66.3	[63.7–68.8]	24	[21.8–26.4]	9.7	[8.3–11.3]	
Believe the effectiveness of the COVID 19 vaccine will be in question due to its fast development							<0.001
Yes	70.5	[68.6–72.3]	17.8	[16.4–19.3]	11.7	[10.4–13.2]	
No	85.7	[82.9–88.0]	7	[5.3–9.2]	7.4	[5.8–9.2]	
Not sure	73.7	[71.3–76.0]	19	[17.0–21.2]	7.3	[6.2–8.5]	
Lost anyone close to you during the lockdown period							0.751
Yes	74.3	[72.5–76.0]	16.2	[14.9–17.7]	9.5	[8.4–10.7]	
No	75.2	[73.1–77.1]	15.4	[13.9–17.1]	9.4	[8.2–10.9]	

### Factors associated with vaccine hesitancy

#### Characteristics of those who were uncertain about taking a COVID-19 vaccination

The adjusted odds of being uncertain about taking a COVID-19 vaccination, compared to being willing to take a vaccination, were significantly higher for the following groups: adults who identified as White (Adjusted Odds Ratio (AOR) = 2.78 [1.80–4.29]) or Colored (AOR = 2.12 [1.57–2.86]) than those who identified as Black African; who had ever refused to take any vaccine (AOR = 1.89 [1.38–2.6]) than those who had not refused; who did not think that or were unsure about whether vaccines were a good way to protect communities from disease (AOR = 4.05 [2.62–6.26], AOR = 3.38 [2.75–4.16], respectively); who felt that their religion or culture would discourage them from getting a COVID-19 vaccination (AOR = 1.39 [1.03–1.9]); who were not confident in the national government’s handling of the pandemic (AOR = 1.43 [1.1–1.86]) compared to those who were very confident; who had concerns about side-effects of the COVID-19 vaccines or were unsure if they had concerns about side-effects (AOR = 6.32 [4.3–9.29] and AOR = 2.92 [1.91–4.46], respectively) than those with no concerns about side-effects; who did not trust or were not sure if they trusted the pharmaceutical industry with developing COVID-19 vaccines (AOR = 3.07 [2.24–4.21] and AOR = 2.53 [1.97–3.25], respectively); who did not trust or were unsure if they trusted the information from their health care provider about the COVID-19 vaccinations (AOR = 1.7 [1.25–2.38] and AOR = 1.89 [1.48–2.42], respectively); who did not think or were unsure about whether there was adequate safety information about the COVID-19 vaccination program (AOR = 1.45 [1.1–1.91] and AOR = 1.44 [1.14–1.82], respectively) and those who believed that the effectiveness of the COVID 19 vaccine would be questionable due to its fast development (AOR = 1.66 [1.2–2.31]) (Table 3).

**Table 3:** Socio-behavioral factors associated with vaccination hesitancy—multiple multinomial regression model.

	Uncertain			Would not take the vaccine		
	AOR	95% CI (AOR)	*p*-value	AOR	95% CI (AOR)	*p*-value
Would take the vaccine (base outcome)						
Gender						
Male	Ref	-	-	Ref	-	-
Female	1.07	[0.89–1.28]	0.478	1.11	[0.86–1.43]	0.429
Other	1.1	[0.62–1.95]	0.751	1.21	[0.49–3.01]	0.683
Age group						
18–29	Ref	-	-	Ref	-	-
30–39	0.76	[0.64–0.89]	0.001	0.65	[0.51–0.82]	<0.001
40–49	0.64	[0.5–0.82]	<0.001	0.67	[0.48–0.94]	0.019
50–59	0.55	[0.37–0.8]	0.002	0.56	[0.35–0.88]	0.013
60–69	0.35	[0.16–0.79]	0.011	0.33	[0.14–0.81]	0.016
70+	0.92	[0.29–2.88]	0.885	0.28	[0.03–2.83]	0.281
Population group						
Black African	Ref	-	-	Ref	-	-
White	2.78	[1.8–4.29]	<0.001	4.3	[2.68–6.92]	<0.001
Colored	2.12	[1.57–2.86]	<0.001	1.73	[1.13–2.63]	0.011
Indian/Asian	1.43	[0.82–2.5]	0.21	1.61	[0.81–3.21]	0.178
Prefer not to answer	0.02	[0–0.05]	<0.001	3.97	[0.71–22.37]	0.118
Province						
Western Cape	Ref	-	-	Ref	-	-
Eastern Cape	1.08	[0.7–1.68]	0.719	1.21	[0.66–2.22]	0.538
Northern Cape	1.21	[0.7–2.11]	0.493	1.68	[0.76–3.74]	0.204
Free State	0.96	[0.63–1.48]	0.866	2.13	[0.98–4.64]	0.057
KwaZulu-Natal	1.22	[0.82–1.84]	0.329	1.29	[0.71–2.36]	0.401
North-West	1.27	[0.84–1.9]	0.257	1.57	[0.85–2.88]	0.147
Gauteng	0.98	[0.7–1.37]	0.898	0.99	[0.6–1.63]	0.976
Mpumalanga	1.29	[0.85–1.95]	0.226	1.11	[0.6–2.05]	0.734
Limpopo	1.11	[0.72–1.7]	0.635	0.98	[0.47–2.06]	0.967
Community type						
City	Ref	-	-	Ref	-	-
Suburb	0.84	[0.54–1.3]	0.44	1.25	[0.79–2]	0.341
Township	1.12	[0.74–1.68]	0.598	1.04	[0.7–1.56]	0.834
Informal settlement	1.35	[0.8–2.29]	0.257	0.91	[0.46–1.77]	0.774
Rural (traditional tribal area)	0.86	[0.55–1.34]	0.514	0.72	[0.44–1.15]	0.168
Farm	1.64	[0.8–3.37]	0.177	1.21	[0.57–2.56]	0.618
Educational attainment						
Less than secondary	Ref	-	-	Ref	-	-
Secondary	1.52	[0.8–2.87]	0.202	0.79	[0.42–1.48]	0.469
Matric	1.59	[0.85–2.97]	0.147	0.73	[0.39–1.36]	0.323
Tertiary	1.49	[0.76–2.91]	0.246	0.54	[0.29–1]	0.052
Employment status						
Employed	Ref	-	-	Ref	-	-
Unemployed	1.02	[0.82–1.27]	0.829	0.86	[0.64–1.16]	0.332
Not in labor force	0.79	[0.48–1.3]	0.349	0.74	[0.42–1.32]	0.311
Ever taken the flu vaccine						
No	Ref	-	-	Ref	-	-
Yes	0.52	[0.41–0.67]	<0.001	0.54	[0.4–0.73]	<0.001
Ever refused to take any vaccine						
No	Ref	-	-	Ref	-	-
Yes	1.89	[1.38–2.6]	<0.001	3.14	[2.25–4.37]	<0.001
Know anyone who has experienced a serious side-effect to any vaccine						
No	Ref	-	-	Ref	-	-
Yes	1.16	[0.96–1.4]	0.129	1.42	[1.1–1.83]	0.007
Perceive vaccines as a good way to protect communities from disease						
Yes	Ref	-	-	Ref	-	-
No	4.05	[2.62–6.26]	<0.001	23.86	[13.94–40.84]	<0.001
Not sure	3.38	[2.75–4.16]	<0.001	4.54	[3.32–6.22]	<0.001
Main source of information on the COVID19 vaccine and vaccinations						
Television	Ref	-	-	Ref	-	-
Radio	0.96	[0.72–1.29]	0.792	0.9	[0.58–1.39]	0.627
News sources (print or online)	1.17	[0.87–1.56]	0.295	0.89	[0.6–1.34]	0.585
Government sources	1.08	[0.71–1.65]	0.717	0.84	[0.56–1.25]	0.386
Medical sources	0.92	[0.6–1.42]	0.72	1.24	[0.63–2.47]	0.532
Social media	0.99	[0.78–1.25]	0.945	0.69	[0.5–0.97]	0.032
Other	0.83	[0.49–1.4]	0.481	1	[0.56–1.77]	0.997
Heard conflicting/confusing information about the COVID-19 vaccinations						
No	Ref	-	-	Ref	-	-
Yes	1.21	[0.96–1.52]	0.099	1.09	[0.8–1.48]	0.601
Feel that your religion or culture would discourage you from getting a COVID-19 vaccination						
No	Ref	-	-	Ref	-	-
Yes	1.39	[1.03–1.9]	0.034	1.88	[1.39–2.54]	<0.001
Confidence in how the national government is handling the pandemic						
Very confident	Ref	-	-	Ref	-	-
Moderately confident	0.97	[0.76–1.23]	0.781	0.78	[0.54–1.12]	0.171
Not confident	1.43	[1.1–1.86]	0.007	1.67	[1.17–2.41]	0.005
I have no opinion	1.13	[0.87–1.47]	0.373	1.27	[0.86–1.87]	0.229
Are you concerned about any side-effects related to the COVID 19 vaccines?						
No	Ref	-	-	Ref	-	-
Yes	6.32	[4.3–9.29]	<0.001	2.21	[1.54–3.18]	<0.001
Not sure	2.92	[1.91–4.46]	<0.001	1.34	[0.75–2.37]	0.325
Trust the pharmaceutical industry with developing the COVID 19 vaccine						
Yes	Ref	-	-	Ref	-	-
No	3.07	[2.24–4.21]	<0.001	8.64	[5.92–12.61]	<0.001
Not sure	2.53	[1.97–3.25]	<0.001	2.57	[1.73–3.81]	<0.001
Trust the information from your health care provider about the COVID-19 vaccinations						
Yes	Ref	-	-	Ref	-	-
No	1.73	[1.25–2.38]	0.001	3.61	[2.51–5.19]	<0.001
Not sure	1.89	[1.48–2.42]	<0.001	2.29	[1.69–3.11]	<0.001
Think there is adequate safety information related to the COVID 19 vaccination program						
Yes	Ref	-	-	Ref	-	-
No	1.45	[1.1–1.91]	0.008	1.67	[1.17–2.39]	0.005
Not sure	1.44	[1.14–1.82]	0.003	1.35	[0.91–2]	0.133
Believe the effectiveness of the COVID 19 vaccine will be in question due to its fast development						
No	Ref	-	-	Ref	-	-
Yes	1.66	[1.2–2.31]	0.002	1.27	[0.81–1.98]	0.302
Not sure	1.4	[0.95–2.05]	0.09	0.92	[0.57–1.48]	0.74

In addition, the odds of being uncertain about taking a COVID-19 vaccination, compared to being willing to take a vaccination, were significantly lower for older adults aged 30–39, 40–49, 50–59 and 60–69 years (AOR = 0.76 [0.64–0.89], AOR = 0.64 [0.5–0.82], AOR = 0.55 [0.37–0.8] and AOR = 0.35 [0.16–0.79], respectively) than those aged 18–29; those who preferred not to report their population group (AOR = 0.02 [0–0.05]) than those who identified as Black African and among those who had ever received an influenza vaccination (AOR = 0.52 [0.41–0.67]) than those who had not.

#### Characteristics of those who were unwilling to take a COVID-19 vaccination

The adjusted odds of being unwilling to take a COVID-19 vaccination, compared to being willing to take one, were significantly higher for adults who identified as White or Colored (AOR = 4.3 [2.68–6.92], AOR = 1.73 [1.13–2.63]) than those who identified as Black African; among those who had ever refused to receive any vaccine (AOR = 3.14 [2.25–4.37]); who knew anyone who experienced a serious side-effect to a vaccine (AOR = 1.42 [1.1–1.83]); who did not perceive or were unsure if they perceived vaccines as being a good way to protect communities from diseases (AOR = 23.86 [13.94–40.84] and AOR = 4.54 [3.32–6.22], respectively); who felt religious or cultural discouragement from taking a COVID-19 vaccine (AOR = 1.88 [1.39–2.54]); who expressed no confidence in the national government’s handling of the pandemic (AOR = 1.67 [1.17–2.41]); who had concerns about side-effects of the COVID-19 vaccines (AOR = 2.21 [1.54–3.18]); who did not trust or were unsure if they trusted the pharmaceutical industry with developing the vaccines (AOR = 8.64 [5.92–12.61] and AOR = 2.57 [1.73–3.81], respectively); who did not trust or were unsure if they trusted the information from their health care provider about the COVID-19 vaccines (AOR = 3.61 [2.51–5.19] and AOR = 2.29 [1.69–3.11], respectively); and who did not think there was adequate safety information about the COVID-19 vaccination program (AOR = 1.67 [1.17–2.39]) (Table 3).

Unwillingness to take a COVID-19 vaccination, compared to willingness to take one, were significantly lower for older adults aged 30–39, 40–49, 50–59 and 60–69 years (AOR = 0.65 [0.51–0.82], AOR = 0.67 [0.48–0.94], AOR = 0.56 [0.35–0.88], AOR = 0.33 [0.14–0.81], respectively) than 18–29 year-olds; for those who had ever received an influenza vaccination (AOR = 0.54 [0.4–0.73]); and whose main source of information on the COVID-19 vaccinations were from social media (AOR = 0.69 [0.5–0.97]).

The socio-demographic factors (gender, education, province, community type and employment status) and having heard conflicting information about the COVID-19 vaccinations, which had significant bivariate associations with vaccine hesitancy, were no longer significantly associated with unwillingness and uncertainty about receiving a vaccination in the regression model which adjusted for all the explanatory variables.

## Discussion

The current study identified several socio-behavioral factors associated with vaccine hesitancy among South African adults, including being of younger age, being of the White and Colored population groups, no history of influenza vaccination, previous vaccination refusal, and knowing someone who experienced a serious vaccination side-effect. Other factors such as misperceptions about vaccinations being beneficial in protecting communities, experiencing cultural or religious discouragement from taking a COVID-19 vaccination, no confidence in the government’s handling of the pandemic, and having concerns about side-effects of the COVID-19 vaccinations were also found. The perceived lack of safety information about the COVID-19 vaccinations and lack of trust in the pharmaceutical industry and in the information from health care providers also contributed to vaccine hesitancy. The following items had stronger associations with unwillingness to take a COVID-19 vaccination than with uncertainty to take the vaccinations: previous refusal to receive any vaccination, not perceiving vaccines as beneficial for protecting communities from disease, individuals from the White population group, religious or cultural discouragement, and lack of trust in the pharmaceutical industry and in the information from health care providers. Concerns about effectiveness of the COVID-19 vaccines due to their fast development was associated with being uncertain about taking the vaccine but not with being unwilling. Having concerns about side-effects had stronger associations with uncertainty to take the vaccine than with unwillingness.

As vaccine-hesitant individuals are a heterogeneous group with varying levels of indecision and apprehensions (19), individuals who are uncertain about their intentions to take a COVID-19 vaccination lie more toward the center of the continuum compared to those who are unwilling. These individuals could be viewed as more amenable to changing their vaccination intentions if their apprehensions are sufficiently addressed than those who reported that they would not take a vaccination, thereby providing key opportunities for public health action. Interventions to improve vaccine uptake should address the identified determinants of vaccine hesitancy. However, interventions can be tailored for those uncertain and unwilling to take a COVID-19 vaccination by addressing the respective determinants of these two types of hesitancy. Therefore, addressing concerns about effectiveness, rapid vaccine development and side-effects need to be focused on more intensively for individuals who are more uncertain about their decision to vaccinate. In comparison, reliable information about why vaccines are protective at population-level, addressing the influence of religious or cultural social norms and attitudes on vaccine behavior and addressing issues of trust in pharmaceutics and the health system are important for individuals who are leaning more toward refusing a COVID-19 vaccination. Furthermore, in the case of adamant vaccine refusers, public health programs should seek to curtail the effect of their anti-vaxxer discourses on other individuals rather than convincing them to change their stance (19).

The finding of higher vaccine hesitancy among young South Africans is consistent with other South African (13, 28, 29) and international studies (15) and evidenced by the lower rates of vaccination coverage among youth in the country (6). Younger individuals tend to be more vaccine hesitant due to their lower risk perceptions regarding severity of COVID-19 in their age group (15). Increasing youth vaccination uptake is important for immunizing communities, due to South Africa having a relatively young population where many youths live in multigenerational households with exposure to older adults. White race was also a predictor of vaccine hesitancy in the United States and Brazil (13, 30, 31). Earlier South African studies confirmed vaccine hesitancy to be highest among White followed by Colored individuals (12, 13). In fact, one study showed that between January to July 2021, vaccine hesitancy increased among white adults, while it decreased for black African adults. It found that concerns about side-effects and vaccine effectiveness were more pronounced among White adults (12).

Favorable experiences with other vaccines can enhance and reinforce trust, self-efficacy and intention regarding taking a COVID-19 vaccination. Having an influenza vaccine and having not refused other vaccines in the past were predictors of vaccine hesitancy. However, as in many developing countries, the majority of the population do not take influenza vaccinations (32). Moreover, Makoae et al. (33) found that South African parents who had never taken an influenza vaccine were significantly less likely to have taken their children for their scheduled vaccinations. Perceptions of vaccines being beneficial for protecting communities was strongly associated with hesitancy, but particularly so with unwillingness to take the vaccination. Lee et al. (34) found that healthcare professionals and community stakeholders who thought that the vaccine could strengthen their immunity against COVID-19, that vaccination was an effective way to prevent COVID-19, that the benefits of COVID-19 vaccination outweighed its harm, and that the vaccine could lower the risk of transmitting the viruses to their family and friends were significantly more likely to get vaccinated. Transparent information, effective science communication strategies and health provider and community discourses are needed to build a greater understanding of why vaccines are needed, how they work in creating herd and population immunity, and how they are developed.

Given that feeling religious or cultural discouragement from taking a COVID-19 vaccine was associated more with unwillingness than uncertainty to take the vaccine; involving religious, traditional and community leaders in vaccine and risk communication and community engagement campaigns can help raise awareness and change social norms (35). Katoto et al. (28) found that many South African community members expressed the desire for religious leaders to be involved in vaccination education programs. Discourses between community members and trusted leaders can help individuals reconcile their vaccination decisions with their values and beliefs. Sharing of personal experiences with vaccines among community members is important.

This study showed that individuals who expressed no confidence in the national government’s handling of the pandemic were more likely to be unwilling or uncertain to take a COVID-19 vaccination. Distrust of government was one of the major predictors of vaccine hesitancy in other South African studies (28, 36). Lack of confidence in government’s handling of the pandemic was also associated with lower engagement in transmission reducing behaviors like social distancing (25). This distrust in government may be due to probes of corruption and fraud related to COVID-19 contracts issued by the South African government (37). Initiatives to address corruption and promote transparent concise communication from government are required.

Mistrust in the pharmaceutical industry, the manufacturers of the COVID-19 vaccines, was a strong predictor of vaccine hesitancy and especially unwillingness to take a COVID-19 vaccine. In 2019, pharmaceutical companies were perceived as the most poorly regarded industry in the United States (38); a perception which is likely to have filtered through to the rest of the world. As such, there has always been public concern over commercial profiteering from the COVID-19 vaccine production (14). Risk and uncertainty are fundamental assumptions of trust (14). Trust in the pharmaceutical industry is therefore inherently linked to concerns over the development process, and hence effectiveness, safety and side-effects.

Health care providers have a responsibility to engage with patients and relay clear, concise and transparent information regarding the vaccines, so that individuals can make informed health decisions. In addition, healthcare providers need to spend sufficient time discussing vaccines with patients, should not deride their concerns, and need to provide satisfactory responses to questions. Mistrust in the information from health providers was associated with vaccine hesitancy in this study. Lower levels of trust in the healthcare system allow individuals to become more sensitive to misinformation about the COVID-19 vaccine (39). In this study, almost half of participants reported being exposed to conflicting information about the COVID-19 vaccines. The finding of lower unwillingness to take a COVID-19 vaccine among those whose main source of vaccine-related information was social media requires further investigation as it is contradictory to previous work (13), where higher vaccine hesitancy was observed among South Africans who had high trust in social media sources. Furthermore there is need to be cognizant of the reality that behavior change cannot be changed by a single intervention such as correct messaging. Therefore, long term multi-level and contextually relevant interventions needs to be designed to address vaccine hesitancy not only for COVID-19, but that straddle across many diseases that requires routine vaccination.

A limitation of this study is that the variables are subject to self-report bias. Furthermore, online surveys are subject to selection bias because individuals who interact more frequently with the internet and smart phones are more likely to participate in an online survey. However, the online surveys were supplemented with telephonic interviews that targeted South Africans in lower-income, high-density areas who have lower internet access, and the data was benchmarked to the general population to increase generalizability of the findings.

## Conclusion

Public health officials and vaccination campaigns need to involve health providers and community and religious leaders in developing and implementing contextually relevant interventions for vaccine hesitancy. Communication campaigns and community engagement strategies need to relaying transparent and concise information about the COVID-19 vaccines, and discuss beliefs, attitudes and barriers and facilitators for vaccine acceptance. It is also critical for health interventions to be designed in such a way that they are culturally relevant and take into consideration the social context of the setting. The health system and public and medical institutions need to demonstrate their trustworthiness to citizens. Ultimately, individuals need to make health care decisions that are aligned with their values (2). In this regard, interventions such as motivational interviewing have been successful in changing vaccination intentions. Comprehensive communication strategies should include issues about side-effects, safety risks, and effectiveness of the vaccines so as to address public concerns about risks of taking a COVID-19 vaccine. This is particularly important for individuals who are uncertain about their vaccine intentions, as it will aid them in making an informed vaccination decision.

## Data availability statement

The raw data supporting the conclusions of this article will be made available by the authors, without undue reservation.

## Ethics statement

Ethical approval to conduct the study was received from the Human Sciences Research Council Research Ethics Committee (HSRC REC) (Protocol number: REC 5/03/20), which is aligned with the principles of the Declaration of Helsinki. Consent for participation was obtained from all participants before they proceeded to the survey questions. Participants were informed of voluntary participation, the anonymity of their responses, and the right to withdraw from the survey at any time.

## Author contributions

RS, SR, SS, and RG: conceptualization and methodology. RS: formal analysis. SR and SS: supervision. TM and IN: validation. RS, RG, IN, and TM: writing original draft. RS, RG, TM, IN, SR, and SS: review and editing of draft manuscript. All authors have read the manuscript and agreed to its submission.

## References

[1] World Health Organization. Ten threats to global health in 2019. (2019). Available at: https://www.who.int/news-room/spotlight/ten-threats-to-global-health-in-2019 (Accessed March 15, 2023).

[2] MacDonald NE. Vaccine hesitancy: definition, scope and determinants. Vaccine. (2015) 33:4161–4. doi: 10.1016/j.vaccine.2015.04.036, PMID: 25896383

[3] Lazarus JV Wyka K White TM Picchio CA Rabin K Ratzan SC. Revisiting COVID-19 vaccine hesitancy around the world using data from 23 countries in 2021. Nat Commun. (2022) 13:3801. doi: 10.1038/s41467-022-31441-x, PMID: 35778396 PMC9247969

[4] Our World in Data. Coronavirus (COVID-19) vaccinations 2023. Available at: https://ourworldindata.org/covid-vaccinations?country=~OWID_WRL.

[5] Coronavirus pandemic (COVID-19). (2020). Available at: https://ourworldindata.org/coronavirus.

[6] South African Department of Health. COVID-19 online resource and news portal (2023). Available at: https://sacoronavirus.co.za/latest-vaccine-statistics/.

[7] Cascini F Pantovic A Al-Ajlouni Y Failla G Ricciardi W. Attitudes, acceptance and hesitancy among the general population worldwide to receive the COVID-19 vaccines and their contributing factors: a systematic review. EClinicalMedicine. (2021) 40:101113. doi: 10.1016/j.eclinm.2021.101113, PMID: 34490416 PMC8411034

[8] Bono SA de Moura F Villela E Siau CS Chen WS Pengpid S. Factors affecting COVID-19 vaccine acceptance: an international survey among low- and middle-income countries. Vaccines. (2021) 9:515. doi: 10.3390/vaccines9050515 34067682 PMC8157062

[9] Grossman VA. The COVID-19 vaccine: why the hesitancy? J Radiol Nurs. (2021) 40:116–9. doi: 10.1016/j.jradnu.2021.02.011, PMID: 33727900 PMC7951560

[10] Caserotti M Girardi P Rubaltelli E Tasso A Lotto L Gavaruzzi T. Associations of COVID-19 risk perception with vaccine hesitancy over time for Italian residents. Soc Sci Med. (2021) 272:113688. doi: 10.1016/j.socscimed.2021.113688, PMID: 33485215 PMC7788320

[11] Roy DN Biswas M Islam E Azam MS. Potential factors influencing COVID-19 vaccine acceptance and hesitancy: a systematic review. PLoS One. (2022) 17:e0265496. doi: 10.1371/journal.pone.0265496, PMID: 35320309 PMC8942251

[12] Runciman C Roberts B Alexander K Bohler-Muller N Bekker M. Willingness to take a COVID-19 vaccine: a research briefing. UJ-HSRC COVID-19 democracy Survey2021. Available at: https://www.uj.ac.za/wp-content/uploads/2022/02/2022-02-02-r5-vaccine-acceptance-and-hesitancy.pdf.

[13] Burger R Köhler T Golos A Buttenheim A English R Tameris M. COVID-19 vaccine hesitancy in South Africa: results from NIDS-CRAMWave 4. (2021). Available at: https://cramsurvey.org/wp-content/uploads/2021/05/3.-Burger-R.-Buttenheim-A.-English-R.-Maughan-Brown-B.-Kohler-T.-_-Tameris-M.-2021.-COVID-19-vaccine-hesitancyin-South-Africa-Results-from-NIDS-CRAM-Wave-4.pdf.

[14] Sapienza A Falcone R. The role of trust in COVID-19 vaccine acceptance: considerations from a systematic review. Int J Environ Res Public Health. (2022) 20:665. doi: 10.3390/ijerph20010665, PMID: 36612982 PMC9819668

[15] Pires C. Global predictors of COVID-19 vaccine hesitancy: a systematic review. Vaccines. (2022) 10:1349. doi: 10.3390/vaccines10081349 36016237 PMC9415631

[16] Viswanath K Bekalu M Dhawan D Pinnamaneni R Lang J McLoud R. Individual and social determinants of COVID-19 vaccine uptake. BMC Public Health. (2021) 21:818. doi: 10.1186/s12889-021-10862-1, PMID: 33910558 PMC8081000

[17] Loomba S de Figueiredo A Piatek SJ de Graff K Larson HJ. Measuring the impact of COVID-19 vaccine misinformation on vaccination intent in the UK and USA. Nat Hum Behav. (2021) 5:337–48. doi: 10.1038/s41562-021-01056-1, PMID: 33547453

[18] Shakeel CS Mujeeb AA Mirza MS Chaudhry B Khan SJ. Global COVID-19 vaccine acceptance: a systematic review of associated social and behavioral factors. Vaccines. (2022) 10:110. doi: 10.3390/vaccines10010110 35062771 PMC8779795

[19] Dubé E MacDonald NE. COVID-19 vaccine hesitancy. Nat Rev Nephrol. (2022) 18:409–10. doi: 10.1038/s41581-022-00571-2, PMID: 35414006 PMC9004449

[20] Wadvalla B-A. How South Africa is dragging its vaccine rollout back from the brink. BMJ. (2021) 374:n1949. doi: 10.1136/bmj.n1949 34426406

[21] Health Justic Initiative. Timeline of key South Africa COVID-19 related developments and health justice Initiative’s engagement with government on vaccine access for south Africa. (2023). Available at: https://healthjusticeinitiative.org.za/vaccine-equity/vaccine-timeline/#:~:text=Phase%201%3A%20February%20%E2%80%93%20April%202021.in%20Phases%201%20and202.

[22] Ramlagan S Shean YL Parker S Trollip K Davids A Reddy SP. Pushing the boundaries: adapting research methodology to document the COVID-19 pandemic from a socio-behavioural perspective in a low/middle level income country: the case of South Africa. Int J Soc Res Methodol. (2022) 25:323–9. doi: 10.1080/13645579.2021.1883538

[23] Reddy SP Sewpaul R Mabaso M Parker S Naidoo I Jooste S. South Africans' understanding of and response to the COVID-19 outbreak: an online survey. S Afr Med J. (2020) 110:894–902. doi: 10.7196/SAMJ.2020.v110i9.14838, PMID: 32880275

[24] Naidoo I Mabaso M Moshabela M Sewpaul R Reddy SP. South African health professionals' state of well-being during the emergence of COVID-19. S Afr Med J. (2020) 110:13104. doi: 10.7196/SAMJ.2020.v110i10.15250, PMID: 33205717

[25] Sewpaul R Mabaso M Dukhi N Naidoo I Vondo N Davids AS. Determinants of social distancing among south Africans from 12 days into the COVID-19 lockdown: a cross sectional study. Front Public Health. (2021) 9:632619. doi: 10.3389/fpubh.2021.632619, PMID: 34109143 PMC8180596

[26] Ipsos. Coronavirus: opinion and reaction results from a multi-country poll. Paris, France: Ipsos (2020).

[27] Statistics South Africa. Mid-year population estimates. Pretoria: Statistics South Africa (2021).

[28] Katoto P Parker S Coulson N Pillay N Cooper S Jaca A. Predictors of COVID-19 vaccine hesitancy in south African local communities: the VaxScenes study. Vaccines. (2022) 10:353. doi: 10.3390/vaccines10030353 35334991 PMC8951818

[29] Kollamparambil U Oyenubi A Nwosu C. COVID19 vaccine intentions in South Africa: health communication strategy to address vaccine hesitancy. BMC Public Health. (2021) 21:2113. doi: 10.1186/s12889-021-12196-4, PMID: 34789201 PMC8596859

[30] Hao F Shao W. Understanding the influence of political orientation, social network, and economic recovery on COVID-19 vaccine uptake among Americans. Vaccine. (2022) 40:2191–201. doi: 10.1016/j.vaccine.2022.02.066, PMID: 35227522 PMC8860708

[31] Moore DCBC Nehab MF Camacho KG Reis AT Junqueira-Marinho MF Abramov DM. Low COVID-19 vaccine hesitancy in Brazil. Vaccine. (2021) 39:6262–8. doi: 10.1016/j.vaccine.2021.09.013, PMID: 34535318 PMC8421107

[32] Solanki G Cornell M Lalloo R. Uptake and cost of influenza vaccines in a private health insured south African population. S Afr J Infect Dis. (2018) 33:a138. doi: 10.4102/sajid.v33i5.138 PMC7015168 32051821

[33] Makoae M Mokhele T Naidoo I Sifunda S Sewpaul R. Determinants of parents taking their children for scheduled vaccinations during COVID-19 pandemic in South Africa. Vaccine. (2023) 11:389. doi: 10.3390/vaccines11020389, PMID: 36851266 PMC9963815

[34] Lee RLT Chien WT Stubbs M Cheng WLS Chiu DCS Fung KHK. Factors associated with COVID-19 vaccine acceptance among healthcare professionals and community stakeholders in Hong Kong: a cross-sectional study. Int J Environ Res Public Health. (2022) 19:14499. doi: 10.3390/ijerph192114499, PMID: 36361376 PMC9656568

[35] Volpp KG Loewenstein G Buttenheim AM. Behaviorally informed strategies for a national COVID-19 vaccine promotion program. JAMA. (2021) 325:125–6. doi: 10.1001/jama.2020.24036, PMID: 33315079 PMC9014824

[36] Engelbrecht M Heunis C Kigozi G. COVID-19 vaccine hesitancy in South Africa: lessons for future pandemics. Int J Environ Res Public Health. (2022) 19:6694. doi: 10.3390/ijerph19116694, PMID: 35682278 PMC9180246

[37] Reuters. South African corruption probe flags COVID contracts worth $137 million. (2022). Available at: https://www.reuters.com/world/africa/safrican-corruption-probe-flags-covid-contracts-worth-137-million-2022-01-25/

[38] McCarthy J. Big pharma sinks to the bottom of US industry rankings. Washington, DC: Gallup Inc (2019).

[39] Wang C Han B Zhao T Liu H Liu B Chen L. Vaccination willingness, vaccine hesitancy, and estimated coverage at the first round of COVID-19 vaccination in China: a national cross-sectional study. Vaccine. (2021) 39:2833–42. doi: 10.1016/j.vaccine.2021.04.020, PMID: 33896661 PMC8043613

