# Supplementary material for: Vaccine hesitancy and related factors among South African adults in 2021: unpacking uncertainty versus unwillingness
Source: Front Public Health. 2023 Nov 3;11:1233031. doi: 10.3389/fpubh.2023.1233031 (PMC10654970; doi:10.3389/fpubh.2023.1233031)
Supplement: Supplementary file 1 [file Table_1.DOCX]

Supplementary Material

Vaccine hesitancy and related factors among South African adults in 2021: unpacking uncertainty versus unwillingness

Authors: Sewpaul R*, Sifunda S, Gaida R, Mokhele T, Naidoo I, Reddy SP.

**Selected Questions**

|  | **When available, would you take the COVID 19 vaccine?** | |
| --- | --- | --- |
| Yes **DEFINITELY**, I would take the vaccine | | 0 |
| Yes **PROBABLY**, I would take the vaccine | | 1 |
| I am uncertain at this stage | | 2 |
| No, **PROBABLY** I would NOT take the vaccine | | 3 |
| No **DEFINITELY**, I would NOT take the vaccine | | 4 |

|  | **How old were you at your last birthday? (in years)** |  |  |
| --- | --- | --- | --- |

|  | **Do you subscribe to any of the following groups?** | Female | Male | Other |
| --- | --- | --- | --- | --- |
|  |  | 1 | 2 | 3 |

|  | **With which of these population groups do you identify?** | | | | |
| --- | --- | --- | --- | --- | --- |
| Black African | | White | Coloured | Indian/Asian | Prefer not to answer |
| 1 | | 2 | 3 | 4 | 5 |

|  | **What best describes the type of community you reside in?** | |
| --- | --- | --- |
| City | | 1 |
| Suburb | | 2 |
| Township | | 3 |
| Informal settlement | | 4 |
| Rural (Traditional tribal area) | | 5 |
| Farm | | 6 |

|  | **What is the highest educational level that you have obtained?** | |
| --- | --- | --- |
| No, formal education | | 0 |
| Primary | | 1 |
| Secondary | | 2 |
| Matric | | 3 |
| Tertiary | | 4 |

|  | **How would you describe your present employment situation?** | |
| --- | --- | --- |
| Employed – full time (fixed salary per month) | | 1 |
| Employed – informal sector/ part time (non-fixed salary per month) | | 2 |
| Unemployed | | 3 |
| Home Duties (not looking for work) | | 4 |
| Full-time Student | | 5 |
| Retired | | 6 |
| Self Employed | | 7 |

|  | **Have you ever taken the FLU vaccine?** | |
| --- | --- | --- |
| Yes | | 0 |
| No | | 1 |

|  | **Have you ever PERSONALLY refused to take any vaccine?**  (example the annual Flu Vaccine; Yellow Fever; Typhoid or similar travel related inoculations) | |
| --- | --- | --- |
| Yes | | 0 |
| No | | 1 |

|  | **Do you know anyone who has personally experienced a *serious* side-effects to any**  **vaccine?** | |
| --- | --- | --- |
| Yes | | 0 |
| No | | 1 |

|  | **Do you think vaccines are a good way to protect communities from disease?** | |
| --- | --- | --- |
| Yes | | 0 |
| No | | 1 |
| Not Sure | | 2 |

|  | **Are you concerned about any *side-effects* related to the COVID 19 vaccines?** | |
| --- | --- | --- |
| Yes | | 0 |
| No | | 1 |
| Not Sure | | 2 |

|  | **What is THE MAIN SOURCE INFORMATION you turn to for information about the COVID 19 vaccine and vaccinations?** | **SINGLE SELECTION** |
| --- | --- | --- |
| Local Television | | 0 |
| Satellite Television (Dstv, Openview) | | 1 |
| Radio | | 2 |
| Print Newspapers | | 3 |
| Print Materials from Local Clinic / Doctor/ Medical Facility | | 4 |
| WhatsApp | | 5 |
| Social Media (Excluding WhatsApp) | | 6 |
| News Websites or Mobile Apps | | 7 |
| Government Sources (President; Minister of Health Etc.) | | 8 |
| Spouse or Children | | 9 |
| Personal Doctor / Medical Facility / Community Health Care Workers | | 10 |
| Friends | | 11 |
| Family | | 12 |
| Other Mobile Chat Services | | 13 |
| Email | | 14 |
| Medical Aid Scheme Information | | 15 |
| SMS | | 16 |
| Traditional Healers | | 17 |
| Religious Leaders | | 18 |
| I Am Not Interested In Receiving Information Related To Covid 19 | | 19 |

|  | **Have you heard conflicting OR confusing information related to the COVID 19 vaccine and vaccinations** | |
| --- | --- | --- |
| Yes | | 0 |
| No | | 1 |

|  | **Do you feel that YOUR religion or culture would discourage you from getting a COVID 19 vaccine for yourself or your family?** | |
| --- | --- | --- |
| Yes | | 0 |
| No | | 1 |

|  | **How confident do you feel about the way the following groups are handling the response to the coronavirus threat:** | | | | |
| --- | --- | --- | --- | --- | --- |
| Please select one option per row | | **Very confident** | **Moderately confident** | **Not confident at all** | **I have no opinion** |
| National Government | |  |  |  |  |

|  | **Do you trust the pharmaceutical industry with developing the COVID 19 vaccine?** | |
| --- | --- | --- |
| Yes | | 0 |
| No | | 1 |
| Not Sure | | 2 |

|  | **Will you trust the information your health care provider may give you about the risks and benefits of COVID 19 vaccinations plan?** | |
| --- | --- | --- |
| Yes | | 0 |
| No | | 1 |
| Not Sure | | 2 |

|  | **Do you think there is adequate safety information related to the COVID 19 vaccination plan in South Africa?** | |
| --- | --- | --- |
| Yes | | 0 |
| No | | 1 |
| Not Sure | | 2 |

|  | **Do you believe the effectiveness of the COVID 19 vaccine will be in question because it was developed so fast?** | |
| --- | --- | --- |
| Yes | | 0 |
| No | | 1 |
| Not Sure | | 2 |

|  | **Have you personally lost anyone close to you during the lockdown period? (Irrespective of the cause of death)** | |
| --- | --- | --- |
| Yes | | 1 |
| No | | 2 |
